# Are age, comorbidity, and frailty associated with complications after minimally invasive esophagectomy? A multicenter cohort study

**DOI:** 10.1093/dote/doag039

**Published:** 2026-05-12

**Authors:** Ruben O Wissing, Eric Matthée, Nikolaj S Baranov, Sander Ubels, Camiel Rosman, Frans van Workum, Bastiaan R Klarenbeek

**Affiliations:** Department of Surgery, Radboud university medical center, Nijmegen, Gelderland, the Netherlands; Department of Surgery, Radboud university medical center, Nijmegen, Gelderland, the Netherlands; Department of Surgery, Canisius-Wilhelmina Hospital, Nijmegen, Gelderland, the Netherlands; Department of Surgery, Radboud university medical center, Nijmegen, Gelderland, the Netherlands; Department of Surgery, Radboud university medical center, Nijmegen, Gelderland, the Netherlands; Department of Surgery, Canisius-Wilhelmina Hospital, Nijmegen, Gelderland, the Netherlands; Department of Surgery, Radboud university medical center, Nijmegen, Gelderland, the Netherlands; Department of Surgery, Radboud university medical center, Nijmegen, Gelderland, the Netherlands; Department of Surgery, Canisius-Wilhelmina Hospital, Nijmegen, Gelderland, the Netherlands; Department of Surgery, Radboud university medical center, Nijmegen, Gelderland, the Netherlands

**Keywords:** minimally invasive esophagectomy, esohageal cancer, risk factors, complications, geriatrics, elderly

## Abstract

**Background:**

Perioperative risks in older, comorbid, and frail patients undergoing open esophagectomy are increased, whereas these risks remain uncertain for minimally invasive esophagectomy (MIE). This study investigates the relationship between age, comorbidity, frailty, and complications after MIE.

**Methods:**

Prospective data from the randomized ICAN trial were used, which compared intrathoracic and cervical anastomosis in adult esophageal cancer patients undergoing MIE. Patients were categorized by age (<75 and ≥ 75 years), comorbidity (ASA, Charlson Comorbidity index (CCI), Cumulative Illness Rating Scale for Geriatrics (CIRS-G)), and frailty (TOPICS-MDS ≥0.20). Primary outcome: severe complications (Clavien-Dindo grade ≥ 3a). Secondary outcomes: overall complications, hospital and intensive care unit (ICU) length of stay (LOS), and hospital readmission. Multivariable regression analysis adjusted for gender and anastomosis location.

**Results:**

Among 245 patients, 87 (35.5%) had severe complications. Eighteen (7.3%) were aged ≥75 years, 49 (20%) had ASA ≥3, 41 (16.7%) had CCI ≥2, median CIRS-G score was 3 (IQR 2.0), and 14 patients (5.7%) were frail. In multivariable regression, age, ASA ≥3, CCI ≥2, and CIRS-G were not independently associated with severe complications, overall complications, readmission, hospital or ICU LOS. Multivariable regression analysis for frailty was limited by the small number of frail patients.

**Conclusions:**

Older and comorbid patients did not experience higher rates of severe complications following MIE, suggesting that they may be considered safe candidates for MIE despite their age. When selected according to current clinical practice, age, and comorbidity alone should not rule out surgical consideration.

## INTRODUCTION

Esophageal cancer ranks as the sixth leading cause of cancer-related death in Western countries, with its incidence increasing.^1^ The cornerstone for curative treatment for esophageal cancer is esophagectomy, which carries a significant risk of postoperative complications and mortality.^2^ Minimally Invasive Esophagectomy (MIE) has demonstrated a reduction in postoperative complications compared to open esophagectomy while maintaining oncological safety.^3–5^ And thus has been widely adopted as a standard approach for the treatment of esophageal cancer patients. However, the safety of MIE for patients aged ≥75 years remains unclear, given their exclusion from many randomized controlled trials.^3^^,^^4^ This leads to daily challenges in selecting patients for MIE, especially considering that more than 30% of patients diagnosed with esophageal cancer are aged ≥75 years.^6^

Despite the advantages of MIE, a substantial proportion of older, comorbid and frail patients with potentially curable esophageal cancer does not undergo surgery, in contrast to younger patients and those without comorbidities or frailty.^6^ Recent studies showed conflicting results regarding the association between comorbidity and frailty with postoperative outcomes following esophagectomy.^7–15^ Most of these studies included open esophagectomies or were performed retrospectively. Notably, no studies investigating perioperative risks after MIE have incorporated focused geriatric frailty scores, which offer a more comprehensive assessment of older patients with comorbidities or frailty.^16^^,^^17^

This study uses the prospectively collected dataset from the ICAN trial, in which patients were not excluded solely on the basis of age, comorbidities, and frailty but were selected according to current clinical practice. The current objective is to explore the association between age (≥75 years), comorbidity, frailty, and the occurrence of severe complications following MIE for esophageal cancer. It was hypothesized that age, comorbidity, and frailty were associated with an increased occurrence of severe complications after MIE for esophageal cancer.

## METHODS

### Study design

For this multicenter cohort study, all prospectively collected patient data from the randomized controlled ICAN trial were used. No patients were excluded from the current analysis. The ICAN trial compared anastomotic leak rates between intrathoracic and cervical anastomosis after MIE.^18^ The study protocol had been previously approved by the institutional review board of the Radboud university medical center and all participating centers, and written informed consent was obtained from all patients before randomization.

### Patient cohort

All patients ≥18 years with primary esophageal cancer who underwent curative, transthoracic (hybrid or total) MIE in an elective setting between April 2016 and January 2020 were included from nine high-volume esophageal cancer centers (minimally 30 resections per year) in the Netherlands. Patients who had undergone previous major gastric or major thoracic surgery, or with a second, prognosis determining malignant condition were excluded. No older patients were excluded. A total of 15 patients did not undergo MIE and were excluded from the ICAN trial, as presented in the previously published flow diagram.^18^ In accordance with national guidelines, patients underwent perioperative chemotherapy, neoadjuvant chemoradiotherapy, or no (neo)adjuvant therapy.^18^

### Outcome measures

The primary outcome of current analysis was severe complications, defined as complications requiring surgical, endoscopic or radiologic intervention (Clavien-Dindo ≥3a).^19^

Secondary outcomes were overall postoperative complications (i.e. regardless of Clavien-Dindo), hospital length of stay (defined as the number of days from hospital admission to discharge), intensive care unit (ICU) length of stay (defined as the number of days from initial ICU admission to discharge to the ward), and hospital readmission rate (defined as any readmission to a primary or secondary hospital within 30 days of discharge).

### Study parameters

#### Patient factors

An age threshold of 75 years was chosen, as previous landmark studies (TIME, MIRO) excluded patients aged ≥75 years.^3^^,^^4^ Comorbidity was measured by three different classifications; the American Society of Anaesthesiologists (ASA) classification, the Charlson Comorbidity Index, and the Cumulative Illness Rating Scale for Geriatrics (CIRS-G).^20–22^

Frailty was measured using The Older Persons and Informal Caregivers Survey Minimum Data Set (TOPICS-MDS) resulting in a score between 0 and 1.^17^ Based on previous literature, a cut-off score ≥ 0.20 was used to indicate frailty.^23^

#### Postoperative outcomes

All postoperative complications were classified according to the Clavien-Dindo classification for surgical complications and transformed into the Comprehensive Complication Index as a measure for overall postoperative complications.^19^^,^^24^ Corresponding to the ICAN trial protocol and the Esophagectomy Complications Consensus Group, postoperative complications were anastomotic leak, pulmonary complications, cardiac complications, and other specified complications occurring within 30 days after MIE.^25^^,^^26^ Further details on postoperative complications are available in Supplement 1.

### Statistical analysis

All statistical analyses were conducted using IBM SPSS Statistics Version 25.^27^ Missing data were handled using pairwise deletion to maximize data availability, as no substantial missing data were observed. Continuous data were described using medians and interquartile ranges (IQRs). *P* values were obtained through Mann–Whitney U tests or Kruskal-Wallis tests, as appropriate. Categorial data were described using frequencies and percentages. *P* values were obtained by using chi-square tests or Fisher’s Exact tests, as appropriate. *P* values <0.05 were considered significant.

**Table 1:** Patient characteristics

**Characteristics, *n* (%)**	**Total (N = 245)**	**Age**		**ASA** ^a^		**Charlson Comorbidity Index**			**TOPICS-MDS frailty index** ^**c**^	
		**<75 years (*n* = 227)**	**≥75 years (*n* = 18)**	**≤2 (*n* = 189)**	**≥3 (*n* = 49)**	**0 (*n* = 161)**	**1 (*n* = 43)**	**≥2 (*n* = 41)**	**No Frailty (*n* = 213)**	**Frailty (*n* = 14)**
**Age, median (IQR), years**	67.2 (9.9)	66.7 (9.6)	78.5 (3.7)	66.7 (10.1)	69.6 (8.6)	66.7 (10.2)	65.5 (8.0)	70.3 (6.9)	67.2 (10.1)	67.4 (5.7)
**Gender**										
Male	190 (77.6)	181 (79.7)	9 (50.0)	148 (78.3)	37 (75.5)	126 (78.3)	32 (74.4)	32 (78.0)	169 (79.3)	6 (42.9)
Female	55 (22.4)	46 (20.3)	9 (50.0)	41 (21.7)	12 (24.5)	35 (21.7)	11 (25.6)	9 (22.0)	44 (20.7)	8 (57.1)
**ASA** ^a^										
≤2	189 (79.4)	178 (80.5)	11 (64.7)			136 (87.7)	31 (73.8)	22 (53.7)	163 (79.1)	10 (71.4)
≥3	49 (20.6)	43 (19.5)	6 (35.3)			19 (12.3)	11 (26.2)	19 (46.3)	43 (20.9)	4 (28.6)
Missing	7 (2.9)									
**Charlson Comorbidity Index**										
0	161 (65.7)	148 (65.2)	13 (72.2)	136 (72.0)	19 (38.8)				138 (64.8)	8 (57.1)
1	43 (17.6)	42 (18.5)	1 (5.6)	31 (16.4)	11 (22.4)				40 (18.8)	1 (7.1)
≥2	41 (16.7)	37 (16.3)	4 (22.2)	22 (11.6)	19 (38.8)				35 (16.4)	5 (35.7)
**CIRS-G, median (IQR)** ^b^	3 (2.0)	3 (3.0)	3.5 (3.3)	2 (3.0)	4.5 (3.0)	2 (2.0)	4 (3.0)	5 (3.3)	3 (3.0)	4 (4.5)
Missing, *n* (%)	10 (4.1)									
**TOPICS-MDS frailty index** ^**c**^										
Frailty	14 (5.7)	13 (6.2)	1 (6.3)	10 (5.8)	4 (8.5)	8 (5.5)	1 (2.4)	5 (12.5)		
Missing	18 (7.3)									
**Tumor type**										
Adenocarcinoma	217 (88.6)	201 (88.5)	16 (88.9)	167 (88.4)	43 (87.8)	141 (87.6)	38 (88.4)	38 (92.7)	188 (88.3)	12 (85.7)
Squamous cell carcinoma	19 (7.8)	17 (7.5)	2 (11.1)	15 (7.9)	4 (8.2)	14 (8.7)	2 (4.7)	3 (7.3)	17 (8.0)	2 (14.3)
Other	7 (2.9)	7 (3.1)	0	5 (2.6)	2 (4.1)	6 (3.7)	1 (2.3)	0	6 (2.8)	0
Unknown	2 (0.8)	2 (0.9)	0	2 (1.1)	0	0	2 (4.7)	0	2 (0.9)	0
**Operation type**										
Totally minimally invasive esophagectomy	197 (80.4)	183 (80.6)	14 (77.8)	155 (82.0)	37 (75.5)	131 (81.4)	35 (81.4)	31 (75.6)	175 (82.2)	12 (85.7)
Hybrid minimally invasive esophagectomy	48 (19.6)	44 (19.4)	4 (22.2)	34 (18.0)	12 (24.5)	30 (18.6)	8 (18.6)	10 (24.4)	38 (17.8)	2 (14.3)
**Anastomosis location**										
Cervical	122 (49.8)	113 (49.8)	9 (50.0)	95 (50.3)	24 (49.0)	79 (49.1)	21 (48.8)	22 (53.7)	105 (49.3)	6 (42.9)
Intrathoracic	123 (50.2)	114 (50.2)	9 (50.0)	94 (49.7)	25 (51.0)	82 (50.9)	22 (51.2)	19 (46.3)	108 (50.7)	8 (57.1)

*Note.*  ^a^American Society of Anesthesiologists classification. ^b^Cumulative Illness Rating Scale for Geriatrics. ^c^The Older Persons and Informal Caregivers Survey Minimum Data Set.

Due to the limited number of patients aged ≥75 years, age was used as a continuous variable in regression analysis. The ASA-classification was clustered into ≤2 and ≥ 3, and the Charlson Comorbidity Index was clustered into 0, 1, and ≥ 2. CIRS-G was used as a continuous variable in regression analysis. Frailty (TOPICS-MDS) was not included in regression analysis due to the limited number of frail patients (TOPICS-MDS ≥0.20). Given the low mortality rates and rates per complication, analyses of these outcomes were descriptive and exploratory in nature.

**Table 2:** Postoperative outcomes

**Outcomes, *n* (%)**	**Total (*N* = 245)**	**Age**			**ASA** ^a^			**Charlson Comorbidity Index**				**TOPICS-MDS frailty index** ^**b**^		
		**<75 years (*n* = 227)**	**≥75 years (*n* = 18)**	***P* value**	**≤2 (*n* = 189)**	**≥3 (*n* = 49)**	***P* value**	**0 (*n* = 161)**	**1 (*n* = 43)**	**≥2 (*n* = 41)**	***P* value**	**No Frailty (*n* = 213)**	**Frailty (*n* = 14)**	***P* value**
**Severe complications**	87 (35.5)	81 (35.7)	6 (33.3)	1.00	68 (36.0)	18 (36.7)	1.00	53 (32.9)	16 (37.2)	18 (43.9)	0.41	72 (33.8)	7 (50.0)	0.25
**Comprehensive Complication Index, median (IQR)**	20.9 (35.4)	20.9 (34.6)	20.9 (42.2)	0.71	20.9 (34.2)	24.2 (18.6)	0.04	20.9 (33.5)	20.9 (43.6)	20.9 (44.2)	0.60	20.9 (33.5)	25.2 (27.0)	0.13
**Length of stay, median (IQR), days**														
Hospital	11 (8.0)	11 (8.0)	14 (8.0)	0.36	11 (8.0)	14 (9.0)	0.10	10 (7.0)	11 (9.0)	13 (11.8)	0.28	10 (7.0)	18 (10.5)	0.049
ICU	2 (2.0)	2 (2.0)	1 (1.3)	0.12	2 (2.0)	2 (2.5)	0.40	2 (2.0)	2 (2.0)	2 (3.0)	0.38	2 (2.0)	2 (3.8)	0.88
**Readmission rate**														
Hospital	36 (14.7)	32 (14.1)	4 (22.2)	0.31	24 (12.7)	12 (24.5)	0.07	21 (13.0)	6 (14.0)	9 (22.0)	0.35	32 (15.0)	1 (7.1)	0.70
ICU	33 (13.5)	30 (13.2)	3 (16.7)	0.72	23 (12.2)	10 (20.4)	0.21	20 (12.4)	8 (18.6)	5 (12.2)	0.55	28 (13.1)	3 (21.4)	0.42

*Note.*  ^a^American Society of Anesthesiologists classification. ^b^The Older Persons and Informal Caregivers Survey Minimum Data Set.

For the primary outcome, risk ratios (RRs), 95% confidence intervals (95% CIs), and *P* values were obtained using log-binomial regression analysis.^28^ For continuous secondary outcomes, regression coefficients (*b*’s) and 95% CIs were calculated using linear regression analysis. For categorial secondary outcomes, regression coefficients and 95% CIs were obtained using log-binomial regression analysis.

Initially, univariable regression analyses were performed to estimate unadjusted associations between individual predictors (age, ASA, Charlson Comorbidity Index, CIRS-G) and the primary and secondary outcomes. Covariates were considered relevant when they potentially affected the outcome parameters, and the number of covariates included in the multivariable model was restricted to prevent overfitting: gender and anastomosis location. To adjust for these covariates, multivariable regression analyses (both log-binomial and linear) were conducted incorporating the predictors (age combined with ASA, Charlson Comorbidity Index, and CIRS-G) and primary and secondary outcomes. The comorbidity parameters (ASA, Charlson Comorbidity Index, and CIRS-G) were included in separate models to avoid multicollinearity.

## RESULTS

### Patient and surgical characteristics

Patient and surgical characteristics are presented in Table 1 and Supplement 2. Among the 245 included patients, 122 patients underwent intrathoracic anastomosis and 123 cervical anastomosis. Eighteen patients (7.3%) were aged ≥75 years, 49 (20%) had ASA ≥3, 41 (16.7%) had Charlson Comorbidity Index ≥2, the median CIRS-G score was 3 (IQR 2.0), and only 14 patients (5.7%) were classified as frail (TOPICS-MDS ≥0.20).

### Postoperative outcomes

Postoperative outcomes and rates of complications are shown in Table 2 and Supplement 3. In total, 87 patients (35.5%) had severe complications. The incidence of severe complications was comparable between patients aged ≥75 and patients aged <75 (33.3% vs. 35.7%, *P* = 1.00), as well as between patients with severe and limited comorbidity (ASA ≥3 vs. ≤2: 36.7% vs. 36.0%, *P* = 1.00; Charlson Comorbidity Index ≥2 vs. 0: 43.9% vs. 32.9%, *P* = 0.41). Frail patients more often had severe complications compared to non-frail patients (50.0% vs. 33.8%, *P* = 0.25). The hospital length of stay was comparable between patients aged ≥75 and patients aged <75 (14 vs. 11 days, *P* = 0.36), as well as between patients with severe and limited comorbidity (ASA ≥3 vs. ≤2: 14 vs. 11 days, *P* = 0.10; Charlson Comorbidity Index ≥2 vs. 0: 13 vs. 10 days, *P* = 0.28). Frail patients had a longer hospital length of stay compared to non-frail patients (18 vs. 10 days, *P* = 0.049).

90-day mortality rates were low across all patient groups: age ≥ 75 years: *n* = 2 (11.1%); age < 75 years: *n* = 4 (1.8%); ASA ≥3: *n* = 3 (6.1%), ASA ≤2: *n* = 3 (1.6%); Charlson Comorbidity Index ≥2: *n* = 1 (2.4%), Charlson Comorbidity Index 0: *n* = 4 (2.5%); frailty: *n* = 1 (7.1%), no frailty: *n* = 4 (1.9%).

### Multivariable analysis

Multivariable analysis of severe complications including risk ratios and 95% CIs are presented in Table 3. Age (RR = 1.01, 95% CI [0.98–1.04]), ASA ≥3 (RR = 1.02, 95% CI [0.56–1.83]), the Charlson Comorbidity Index (1: RR = 1.34, 95% CI [0.74–2.42]; ≥2: RR = 1.30, 95% CI [0.71–2.37]), and CIRS-G (RR = 1.03, 95% CI [0.93–1.14]) were not significantly associated with the incidence of severe complications.

**Table 3:** Univariable and multivariable regression model for primary outcome

**Predictors**	**Severe complications**			
	**Univariable RR (95% CI)**	***P* value**	**Multivariable RR (95% CI)**	***P* value**
Age	1.02 (0.99–1.05)	0.26	1.01 (0.98–1.04)	0.62
ASA				
≤2		1 [Reference]		1 [Reference]
≥3	1.02 (0.61–1.72)	0.94	1.02 (0.56–1.83)	0.96
Charlson Comorbidity Index				
0		1 [Reference]		1 [Reference]
1	1.13 (0.65–1.98)	0.67	1.34 (0.74–2.42)	0.34
≥2	1.33 (0.78–2.28)	0.29	1.30 (0.71–2.37)	0.40
CIRS-G	1.03 (0.94–1.14)	0.49	1.03 (0.93–1.14)	0.59

*Note.* Multivariable analyses adjusted for gender and anastomosis location. ASA, Charlson Comorbidity Index, and CIRS-G were included in separate models to avoid multicollinearity. Risk ratios (RR) are significant at *P* < .05 when one is not included in the 95% confidence interval (95% CI). ^*^P < .05. ^**^P < .01. ^***^P < .001.

Multivariable models, including regression coefficients and 95% CIs, of respectively overall postoperative complications, hospital length of stay, ICU length of stay and hospital readmission rate are presented in Table 4. There were no significant associations between age, ASA ≥3, the Charlson Comorbidity Index, CIRS-G and the secondary outcome measures: Comprehensive Complication Index, hospital length of stay, ICU length of stay, and hospital readmission rate.

**Table 4:** Univariable and multivariable regression model for secondary outcomes

**Predictors**	**Comprehensive complication index**		**Hospital length of stay**		**ICU length of stay**		**Hospital readmission rate**	
	**Univariable *b* (95% CI)**	**Multivariable *b* (95% CI)**	**Univariable *b* (95% CI)**	**Multivariable *b* (95% CI)**	**Univariable *b* (95% CI)**	**Multivariable *b* (95% CI)**	**Univariable *b* (95% CI)**	**Multivariable *b* (95% CI)**
Age	0.37 (−0.06–0.81)	0.31 (−0.15–0.76)	0.13 (−0.11–0.37)	0.08 (−0.16–0.33)	0.03 (−0.11–0.17)	0.01 (−0.14–0.16)	−0.01 (−0.06–0.04)	−0.01 (−0.06–0.05)
ASA								
≤2	[Reference]	[Reference]	[Reference]	[Reference]	[Reference]	[Reference]	[Reference]	[Reference]
≥3	7.99 (0.05–15.94)	7.36 (−0.57–15.28)	1.71 (−2.69–6.11)	1.52 (−2.88–5.93)	0.00 (−2.55–2.55)	−0.06 (−2.65–2.53)	0.54 (−0.24–1.32)	0.59 (−0.20–1.38)
Charlson Comorbidity Index								
0	[Reference]	[Reference]	[Reference]	[Reference]	[Reference]	[Reference]	[Reference]	[Reference]
1	5.41 (−3.17–13.99)	5.89 (−2.58–14.36)	3.85 (−0.83–8.53)	4.02 (−0.62–8.67)	1.74 (−0.99–4.48)	1.76 (−1.00–4.51)	−0.01 (−1.00–0.98)	−0.02 (−1.01–0.97)
≥2	5.60 (−3.14–14.34)	4.11 (−4.62–12.84)	2.52 (−2.25–7.29)	1.91 (−2.89–6.71)	1.55 (−1.24–4.34)	1.44 (−1.40–4.27)	0.36 (−0.52–1.23)	0.39 (−0.50–1.28)
CIRS-G	1.23 (−0.22–2.67)	1.04 (−0.42–2.50)	0.57 (−0.22–1.36)	0.49 (−0.32–1.29)	0.26 (−0.20–0.72)	0.24 (−0.24–0.71)	−0.09 (−0.25–0.08)	−0.08 (−0.26–0.09)

*Note.* Multivariable analyses adjusted for gender and anastomosis location. ASA, Charlson Comorbidity Index, and CIRS-G were included in separate models to avoid multicollinearity. Regression coefficients (*b*) are significant at *P* < .05 when zero is not included in the 95% confidence interval (95% CI).

## DISCUSSION

This study found that age, comorbidity, and frailty did not independently increase the rate of severe complications following MIE. Moreover, these factors were not associated with overall postoperative complications, hospital and ICU length of stay, or hospital readmission rates.

Previous literature found conflicting results regarding the relationship between complications and age, comorbidity, and frailty, in patients undergoing esophagectomy. Regarding comorbidity, some previous studies found that comorbidity was associated with more severe complications, however these studies differed from the current study as they included open esophagectomies.^7–10^ Our data does not align with these previous studies, as we did not observe a significant increase of severe complications in patients categorized as severely comorbid according to validated comorbidity measurements.^20–22^ Despite the relatively low comorbidity rates in our dataset, our sample reflects daily practice patient selection, suggesting that appropriately selected patients aged 75 years and older with comorbidities may be viable candidates for MIE.

The study’s findings indicate that selected patients aged 75 years and older, irrespective of comorbidity status, do not exhibit increased complication rates within the group of patients typically considered eligible for surgery. This suggests that, when selected according to current clinical practice, these patients may safely undergo MIE without an increased risk of complications. Unfortunately, no data were gathered regarding the specifics on how patients were selected (e.g. preoperative screening by an anesthesiologist or geriatrician). This study provides prospective insights that age and comorbidity alone should not rule out surgical consideration. However, further research is required to optimize patient selection strategies and criteria.

Clinicians often face a dilemma when balancing potential survival benefits against the risks of complications before recommending surgery, as short-term complications may adversely impact recovery, quality of life, and possibly long-term survival.^29^ Although older and comorbid patients may not experience short-term complications more frequently, the burden of the postoperative course may be greater in this group. This consideration is especially relevant for elderly patients, for whom preservation of quality of life is often most important. Consequently, future studies should assess the impact of each esophageal cancer treatment strategy on quality of life to provide insight into the optimal treatment strategies for these patient populations. Ultimately, future research should incorporate these findings in the development of user-friendly clinical decision tools to enhance risk assessment, patient selection and patient counselling for MIE in daily practice.

The major strength of this study is the utilization of a prospective dataset with standardized outcome measures, prospective data collection, and rigorous data validation procedures.^18^^,^^25^ As a result, the data employed in this study is highly reliable. Furthermore, due to treatment homogeneity and broad inclusion criteria, the current sample closely reflects real-life practice, increasing generalizability of the study results regarding esophageal cancer patients eligible for curative resection.^12^^,^^18^^,^^25^^,^^30^ Some limitations must also be acknowledged. First, the study was not powered for the current objectives, which might explain the non-significant outcomes. Therefore, the absence of an effect cannot be definitively concluded. Specifically for frailty, a post hoc effect size calculation showed that the observed difference in severe complications was statistically undetectable due to the small sample size. As this difference may still be clinically relevant, a larger sample is needed to validate this finding. In addition, the mortality rates were too low to draw meaningful conclusions. Second, because the study cohort exclusively consists of patients enrolled in a prospective minimally invasive surgery trial, the study suffers from selection bias. This sample likely represents healthier esophageal cancer patients indicated for surgery, and may not be generalizable to the broader population of patients undergoing esophagectomy. This may partially explain the observed lack of association. Severely frail patients often receive definitive chemoradiotherapy instead of neoadjuvant chemoradiotherapy followed by surgery.^2^ Nevertheless, the population-based Dutch Upper-GI Cancer Audit (DUCA) and this study have shown comparable proportions of patients aged 75 years and older and patients with comorbidities, showing that the current sample closely reflects patient selection in daily practice.^12^ Third, unmeasured confounders might have influenced the results. For instance, standard physical function tests, which could predict adverse outcomes after surgery, could not be evaluated as our study did not measure this.^31^

## CONCLUSIONS

In this multicenter cohort from the randomized ICAN trial, older and comorbid patients did not experience higher rates of severe complications following MIE. These findings suggest that, when selected according to current clinical practice, such patients may be considered candidates for MIE safely. Our study provides prospective insights that age and comorbidity alone should not rule out surgical consideration.

## Supplementary Material

Supplementary_material_doag039

## Notes

Correspondence during review process: Ruben O. Wissing, Department of Surgery, Radboud University Medical Center, P.O. Box 9101, 6500 HB, Nijmegen, The Netherlands. Email: Ruben.Wissing@radboudumc.nl

Specific author contributions: Ruben Oscar Wissing (Conceptualization, Data curation, Formal analysis, Investigation, Methodology, Project administration, Visualization, Writing—original draft, Writing—review & editing), Eric Matthée (Conceptualization, Data curation, Methodology, Writing—review & editing), Sander Ubels (Conceptualization, Data curation, Formal analysis, Investigation, Methodology, Project administration, Writing—review & editing), Nikolaj Baranov (Conceptualization, Data curation, Formal analysis, Investigation, Methodology, Project administration, Writing—original draft, Writing—review & editing), Camiel Rosman (Conceptualization, Funding acquisition, Investigation, Methodology, Resources, Supervision, Writing—review & editing), Frans van Workum (Conceptualization, Data curation, Funding acquisition, Investigation, Methodology, Resources, Supervision, Validation, Writing—review & editing), Bastiaan R Klarenbeek (Conceptualization, Data curation, Investigation, Methodology, Supervision, Writing—review & editing).

Concept and design: Wissing, Baranov, Ubels, Matthée, Rosman, van Workum, Klarenbeek; Acquisition, analysis, or interpretation of data: Wissing, Baranov, Ubels, Matthée, Rosman, van Workum, Klarenbeek; Drafting of the manuscript: Wissing, Baranov; Critical revision of the manuscript for important intellectual content: Wissing, Baranov, Ubels, Matthée, Rosman, van Workum, Klarenbeek; Statistical analysis: Wissing, Baranov, Ubels; Obtained funding: van Workum, Rosman; Administrative, technical, or material support: Wissing, Baranov, Ubels; Supervision: Rosman, van Workum, Klarenbeek.

Financial support: This study used data of the ICAN trial, which was funded by The Netherlands Organization for Health Research Development Health Care Efficiency Research program (ZonMw; grant 843002607). The funder had no role in the design, conduct, analysis or reporting of the trial and current study.
