# Supplementary material for: Are age, comorbidity, and frailty associated with complications after minimally invasive esophagectomy? A multicenter cohort study
Source: Dis Esophagus. 2026 May 12;39(3):doag039. doi: 10.1093/dote/doag039 (PMC13166868; doi:10.1093/dote/doag039)
Supplement: Supplementary_material_doag039 [file supplementary_material_doag039.docx]

**Supplementary material**

**Supplement 1.** Postoperative Complication Definitions

Anastomotic leak was defined as a full-thickness defect of the anastomosis requiring endoscopic, radiologic or surgical reintervention. Pulmonary complications included pneumonia, pneumothorax requiring drainage, pleural effusion requiring drainage, empyema requiring drainage, tracheobronchial defect, and respiratory failure requiring reintubation. Cardiac complications included supraventricular arrhythmia, ventricular arrhythmia, cardiac decompensation, and myocardial infarction. Other recorded postoperative complications included gastric conduit leakage, gastric conduit necrosis, chyle leakage, mediastinal fluid collection, wound abscess, deep vein thrombosis, pulmonary embolism, cerebrovascular accident, and recurrent laryngeal nerve palsy.

| **Supplement 2.** Operation and Pathology Characteristics Stratified by Frailty | | | |
| --- | --- | --- | --- |
| **Characteristics, *n* (%)** | **Total (N = 245)** | **TOPICS-MDS frailty index^a^** | |
|  |  | **No Frailty (n = 213)** | **Frailty (n = 14)** |
| **Tumor location** |  |  |  |
| Intrathoracic midesophagus | 9 (3.7) | 9 (4.2) | 0 |
| Intrathoracic distal esophagus | 211 (86.1) | 183 (85.9) | 11 (78.6) |
| Gastroesophageal junction | 25 (10.2) | 21 (9.9) | 3 (21.4) |
| **cT stage** |  |  |  |
| T1 | 2 (0.8) | 2 (0.9) | 0 |
| T1a | 2 (0.8) | 2 (0.9) | 0 |
| T1b | 2 (0.8) | 1 (0.5) | 1 (7.1) |
| T2 | 42 (17.1) | 36 (16.9) | 2 (14.3) |
| T3 | 133 (54.3) | 113 (53.1) | 9 (64.3) |
| T4 | 1 (0.4) | 1 (0.5) | 0 |
| T4a | 2 (0.8) | 1 (0.5) | 0 |
| Tx | 61 (24.9) | 57 (26.8) | 2 (14.3) |
| **cN stage** |  |  |  |
| N0 | 113 (46.1) | 98 (46.0) | 6 (42.9) |
| N1 | 84 (34.3) | 71 (33.3) | 5 (35.7) |
| N2 | 35 (14.3) | 33 (15.5) | 1 (7.1) |
| N3 | 4 (1.6) | 3 (1.4) | 1 (7.1) |
| N+ | 6 (2.4) | 5 (2.3) | 1 (7.1) |
| Nx | 3 (1.2) | 3 (1.4) | 0 |
| **Neoadjuvant treatment** |  |  |  |
| Chemoradiotherapy | 235 (95.9) | 204 (98.1) | 13 (92.9) |
| Chemotherapy | 5 (2.0) | 4 (1.9) | 1 (7.1) |
| None | 5 (2.0) | 5 (2.3) | 0 |
| **Configuration of anastomosis** |  |  |  |
| End to end | 50 (20.4) | 41 (19.2) | 4 (28.6) |
| End to side | 151 (61.6) | 136 (63.6) | 5 (35.7) |
| Side to side | 44 (18.0) | 37 (17.3) | 5 (35.7) |
| **Anastomosis technique** |  |  |  |
| Handsewn | 112 (45.7) | 96 (44.9) | 6 (42.9) |
| Stapled | 133 (54.3) | 118 (55.1) | 8 (57.1) |
| **Conversion** |  |  |  |
| To laparotomy | 8 (3.3) | 7 (3.3) | 0 |
| To thoracotomy | 7 (2.9) | 7 (3.3) | 0 |
| **Operating time, median (IQR), min** | 270 (96.5) | 270 (90.0) | 242.5 (102.0) |
| **Blood loss, median (IQR), mL** | 100 (200.0) | 100 (150.0) | 75 (218.0) |
| **Lymph nodes, median (IQR)** |  |  |  |
| Retrieved | 22 (10.0) | 23 (9.5) | 19.5 (15.3) |
| Positive | 0 (1.0) | 0 (1.0) | 1 (2.3) |
| **Radical resection** | 242 (98.8) | 211 (99.0) | 14 (100.0) |
| *Note.* ^a^ The Older Persons and Informal Caregivers Survey Minimum Data Set. | | | |

| **Supplement 3.** Mortality and Complication Rates | | | | | | | | | | |
| --- | --- | --- | --- | --- | --- | --- | --- | --- | --- | --- |
| **Outcomes, *n* (%)** | **Total (N = 245)** | **Age** | | **ASA**^a^ | | **Charlson Comorbidity Index** | | | **TOPICS-MDS frailty index^b^** | |
|  |  | **<75 years (n = 227)** | **≥75 years (n = 18)** | **≤2 (n = 189)** | **≥3 (n = 49)** | **0 (n = 161)** | **1 (n = 43)** | **≥2 (n = 41)** | **No Frailty (n = 213)** | **Frailty (n = 14)** |
| **Mortality** |  |  |  |  |  |  |  |  |  |  |
| In-hospital | 4 (1.6) | 3 (1.3) | 1 (5.6) | 2 (1.1) | 2 (4.1) | 2 (1.2) | 1 (2.3) | 1 (2.4) | 2 (0.9) | 1 (7.1) |
| 30-day | 4 (1.6) | 3 (1.3) | 1 (5.6) | 2 (1.1) | 2 (4.1) | 2 (1.2) | 1 (2.3) | 1 (2.4) | 2 (0.9) | 1 (7.1) |
| 90-day | 6 (2.4) | 4 (1.8) | 2 (11.1) | 3 (1.6) | 3 (6.1) | 4 (2.5) | 1 (2.3) | 1 (2.4) | 4 (1.9) | 1 (7.1) |
| **Anastomotic leak** |  |  |  |  |  |  |  |  |  |  |
| Requiring reintervention | 54 (22) | 52 (22.9) | 2 (11.1) | 46 (24.3) | 8 (16.3) | 27 (16.8) | 13 (30.2) | 14 (34.1) | 47 (22.1) | 3 (21.4) |
| Total | 57 (23.3) | 55 (24.2) | 2 (11.1) | 49 (25.9) | 8 (16.3) | 29 (18.0) | 14 (32.6) | 14 (34.1) | 49 (23.0) | 4 (28.6) |
| **Gastric conduit** |  |  |  |  |  |  |  |  |  |  |
| Leakage | 1 (0.4) | 1 (0.4) | 0 | 1 (0.5) | 0 | 1 (0.6) | 0 | 0 | 1 (0.5) | 0 |
| Necrosis | 3 (1.2) | 2 (0.9) | 1 (5.6) | 1 (0.5) | 2 (4.1) | 2 (1.2) | 0 | 1 (2.4) | 2 (0.9) | 0 |
| **Pulmonary complications** |  |  |  |  |  |  |  |  |  |  |
| Pneumonia | 37 (15.1) | 35 (15.4) | 2 (11.1) | 25 (13.2) | 12 (24.5) | 24 (14.9) | 5 (11.6) | 8 (19.5) | 33 (15.5) | 4 (28.6) |
| Pneumothorax requiring drainage | 10 (4.1) | 10 (4.4) | 0 | 7 (3.7) | 3 (6.1) | 5 (3.1) | 2 (4.7) | 3 (7.3) | 10 (4.7) | 0 |
| Pleural effusion requiring drainage | 38 (15.5) | 37 (16.3) | 1 (5.6) | 26 (13.8) | 11 (22.4) | 24 (14.9) | 6 (14.0) | 8 (19.5) | 30 (14.1) | 3 (21.4) |
| Empyema requiring drainage | 10 (4.1) | 10 (4.4) | 0 | 8 (4.2) | 2 (4.1) | 6 (3.7) | 4 (9.3) | 0 | 8 (3.8) | 0 |
| Tracheobronchial defect | 1 (0.4) | 1 (0.4) | 0 | 1 (0.5) | 0 | 0 | 1 (2.3) | 0 | 1 (0.5) | 0 |
| Respiratory failure requiring reintubation | 23 (9.4) | 21 (9.3) | 2 (11.1) | 17 (9.0) | 6 (12.2) | 10 (6.2) | 5 (11.6) | 8 (19.5) | 20 (9.4) | 1 (7.1) |
| **Chyle leakage** | 20 (8.2) | 20 (8.8) | 0 | 16 (8.5) | 3 (6.1) | 13 (8.1) | 4 (9.3) | 3 (7.3) | 16 (7.5) | 1 (7.1) |
| **Mediastinal fluid collection** | 7 (2.9) | 7 (3.1) | 0 | 5 (2.6) | 2 (4.1) | 2 (1.2) | 2 (4.7) | 3 (7.3) | 7 (3.3) | 0 |
| **Wound abscess** | 14 (5.7) | 14 (6.2) | 0 | 8 (4.2) | 6 (12.2) | 4 (2.5) | 3 (7.0) | 7 (17.1) | 12 (5.6) | 2 (14.3) |
| **Cardiovascular complications** |  |  |  |  |  |  |  |  |  |  |
| Myocardial infarction | 0 | 0 | 0 | 0 | 0 | 0 | 0 | 0 | 0 | 0 |
| Supraventricular arrhythmia | 42 (17.1) | 40 (17.6) | 2 (11.1) | 30 (15.9) | 12 (24.5) | 25 (15.5) | 10 (23.3) | 7 (17.1) | 35 (16.4) | 1 (7.1) |
| Ventricular arrhythmia | 3 (1.2) | 3 (1.3) | 0 | 1 (0.5) | 2 (4.1) | 1 (0.6) | 2 (4.7) | 0 | 3 (1.4) | 0 |
| Cardiac decompensation | 3 (1.2) | 3 (1.3) | 0 | 2 (1.1) | 1 (2.0) | 0 | 1 (2.3) | 2 (4.9) | 2 (0.9) | 1 (7.1) |
| Deep vein thrombosis | 2 (0.8) | 2 (0.9) | 0 | 2 (1.1) | 0 | 2 (1.2) | 0 | 0 | 1 (0.5) | 0 |
| Pulmonary embolism | 3 (1.2) | 3 (1.3) | 0 | 2 (1.1) | 1 (2.0) | 3 (1.9) | 0 | 0 | 3 (1.4) | 0 |
| Cerebrovascular accident | 1 (0.4) | 1 (0.4) | 0 | 1 (0.5) | 0 | 1 (0.6) | 0 | 0 | 1 (0.5) | 0 |
| **Recurrent laryngeal nerve palsy** | 9 (3.7) | 6 (2.6) | 3 (16.7) | 6 (3.2) | 3 (6.1) | 4 (2.5) | 2 (4.7) | 3 (7.3) | 8 (3.8) | 0 |
| *Note.* ^a^ American Society of Anesthesiologists classification. ^b^ The Older Persons and Informal Caregivers Survey Minimum Data Set. | | | | | | | | | | |
